# Study on the Mechanical Properties of Bionic Coupling Layered B_4_C/5083Al Composite Materials

**DOI:** 10.3390/ma11050680

**Published:** 2018-04-26

**Authors:** Qian Zhao, Yunhong Liang, Qingping Liu, Zhihui Zhang, Zhenglei Yu, Luquan Ren

**Affiliations:** 1 The Key Laboratory of Bionic Engineering, Ministry of Education, Jilin University, Changchun 130025, China; qianzhao16@mails.jlu.edu.cn (Q.Z.); zhzh@jlu.edu.cn (Z.Z.); zlyu@jlu.edu.cn (Z.Y.); lqren@jlu.edu.cn (L.R.); 2 State Key Laboratory of Automotive Simulation and Control, Jilin University, Changchun 130025, China

**Keywords:** shell, bionic design, coupling layered structure, mechanical property

## Abstract

Based on microstructure characteristics of *Meretrix lusoria* shell and *Rapana venosa* shell, bionic coupling layered B_4_C/5083Al composites with different layered structures and hard/soft combination models were fabricated via hot pressed sintering. The simplified bionic coupling models with hard and soft layers were similar to layered structure and hardness tendency of shells, guiding the bionic design and fabrication. B_4_C/5083Al composites with various B_4_C contents and pure 5083Al were treated as hard and soft layers, respectively. Hot pressed sintering maintained the designed bionic structure and enhanced high bonding strength between ceramics and matrix. Compared with B_4_C/5083Al composites, bionic layered composites exhibited high mechanical properties including flexural strength, fracture toughness, compressive strength and impact toughness. The hard layers absorbed applied loads in the form of intergranular fracture. Besides connection role, soft layers restrained slabbing phenomenon and reset extension direction of cracks among layers. The coupling functions of bionic composites proved the feasibility and practicability of bionic fabrication, providing a new method for improvement of ceramic/Al composite with properties of being lightweight and high mechanical strength.

## 1. Introduction

With the development of science and industrial technology, the applied conditions of high temperature, speed and load increase the requirements of combination properties of materials. In fields of aviation, aerospace, weapons, vehicles and ships, besides the low density, high level of strength and high level of toughness, materials should have an impact resistance property, which enhanced difficulties of design and fabrication of engineering materials. Therefore, how to improve the corresponding mechanical properties while being lightweight is attractive but still challenging. As a candidate for engineering materials, a ceramic reinforced Al matrix composite combined a high elastic modulus, strength and hardness of ceramics and the low density and high ductility of Al [1,2,3,4], leading to the characteristics of being lightweight and having a high level of strength of ceramic/Al composites. Besides the increase of specific strength and modulus, the addition of ceramics including TiC, TiB_2_, SiC and B_4_C [5,6,7,8,9] decreases the tenacity of an Al matrix, resulting in the possibility of brittle rupture. The corresponding shortcomings restrict the practical application of ceramic/Al composites.

Many kinds of technologies have been adopted to solve the problem of being lightweight, having a high level of strength and high ductility of ceramic/Al composites, such as matrix modification [10,11], interfaces modification [12] and improvement of the fabrication method [1,13,14]. A second metal was used to add into material systems, which formed an intermetallic compound of Al-Metal. The hardness of Al-Metal fabricated via matrix modification was higher and lower than an Al matrix and ceramics, respectively, forming a hardness gradient transition in ceramic/Al composites [15]. Interfaces modification improved the distribution of ceramics in the matrix and enhanced bonding strength between ceramics and the matrix, which reduced the exfoliation of ceramics during the mechanical tests process [16]. Many kinds of methods have been improved and used to fabricate ceramic/Al composites with high mechanical properties, such as stirring casting technique [17], melt infiltration process [18], and so on. Compared with the other methods, hot pressed sintering [1,19] solved the limitations of compactness and enhanced mechanical properties further. These technologies focused on resolving combination abilities of a high level of strength and a high level of toughness of ceramic/Al composites. However, the achieved improvements can not satisfy the rigorous demands of practical application.

The bionic investigation promoted the development of science technology. Many kinds of biology in nature owned properties of being lightweight, having a high level of strength and a high level of toughness, which provided significant references for improving properties of ceramic/Al composites. Due to an ordered assembly of mineral tiles with an approximate thickness of 0.5 mm and diameter of 10 mm, fracture toughness of abalone (*Haliotis rufescens*) nacre [20] was superior to that of monolithic calcium carbonate. Exoskeleton of the arthropod [21] with mineralized chitin layers was effective in crack arrest property. Toucan beak [22] owned the porous interior with a central void region, which decreased the weight of beaks and resisted flexure stresses without buckling. In our previous studies [23], the effect of microstructure on mechanical properties of the *white clam* shell was investigated. The layered structure and hardness gradient built a material base for flexural strength, compressive strength and crack arrest property. Based on a *white clam* shell, we designed and fabricated a kind of impact resistance material successfully [24], exhibiting the feasibility of bionic fabrication. The biology with being lightweight, having a high level of strength and a high level of toughness provided a solution for structure design and combination between being lightweight and having a high level of mechanical properties of ceramic/Al composite materials.

The properties of high corrosion resistance, weldability and strength resulted in the wide use of 5083Al in practical application. Because of the good chemical and thermal stability, lower density and higher elastic modulus, B_4_C was an attractive reinforcement among various ceramic particulates. Therefore, B_4_C/5083Al composite material was chosen for bionic ceramic/Al composite design. In this paper, we investigated structure characteristics of two typical shells of *Meretrix lusoria* and *Rapana venosa*. According to the design of bionic models, B_4_C/5083Al composites with different B_4_C contents were synthesized via hot pressed sintering to fabricated bionic layered *Meretrix lusoria* shell and *Rapana venosa* shell materials. The fabricated bionic composites owned were lightweight, having a high level of strength and a high level of toughness and provided a new idea and method for the practical application of ceramic/Al composite in engineering.

## 2. Experimental Procedure

### 2.1. Materials and Fabrication of Bionic Layered Composite Materials

The *Meretrix lusoria* and *Rapana venosa* were purchased in the aquatic product market of Changchun, China. After removing soft tissues, the *Meretrix lusoria* and *Rapana venosa* shells were rinsed by distilled water, and dried at room temperature for three days. The breaking fragments obtained from shells that were close to the wide marginal edge were used for microstructure observation via scanning electron microscopy (SEM) (Model Evo18 Carl Zeiss, Oberkochen, Germany) together with energy-dispersive spectrometry (EDS) (Model Oxford Instruments, Oxford, UK). The starting materials were purchased from commercial powders of aluminum (99.5% purity, ~48 µm) and boron carbide (99.9% purity, ~13 µm), respectively. B_4_C contents with 10, 20 and 30 wt % of the total weight of the mixture were chosen to adjust hardness values of B_4_C/5083Al composites. The powders were mixed in a stainless steel container using stainless-steel balls at a low speed (~35 rpm) for 8 h to ensure homogeneity. According to a layered structure and hardness distribution pattern of *Meretrix lusoria* shell and *Rapana venosa* shell, B_4_C/5083Al systems with different B_4_C contents and pure 5083 Al were laid into a graphite die with 85 mm in diameter alternately and pressed into a precast block. B_4_C/5083Al layers occupied the outer, middle and inner layers, respectively. Pure 5083Al layers were arranged between arbitrary two B_4_C/5083Al layers, forming the layered pattern. The graphite die with compact was put into an intermediate frequency furnace under air. The layered cylindrical compacts were heated to 650 °C. The temperature of graphite die was measured by an infrared temperature measuring sensor (Asmik, Hangzhou, China). Then, the graphite die underwent heat preservation for 10 min. After the temperature of the graphite die decreases to 550 °C and is maintained for 10 min, the load of 3 t was applied on a graphite die to increase the compactness of the composite materials. After pressure preservation of 5 min and cooling progress, the composite was prepared for metallographic and mechanical experiments. In order to satisfy dimension demand of mechanical tests and enhance the practical fabrication feasibility, bionic layered composite materials with Φ85 mm × 6 mm and Φ85 mm × 10 mm were prepared, respectively. The homogeneous B_4_C/5083Al composite was fabricated under the same preparation conditions. The phase component was identified using X-ray diffraction (XRD) (Model D/Max 2500PC, Rigaku, Tokyo, Japan).

### 2.2. Metallographic and Mechanical Characteristics

#### 2.2.1. Metallographic Characteristic

The bionic layered composite materials with dimension of 10 mm × 10 mm × 10 mm (Length × Width × Thickness) were ground to get a smooth surface. After etching by Keller’s reagent (1 vol. % HF + 1.5 vol. % HCl + 2.5 vol. % HNO_3_ + 95 vol. % H_2_O), the metallographic surfaces were observed via SEM.

#### 2.2.2. Microhardness

The microhardness along thickness direction of bionic layered composite material was measured by a microhardness testing machine with applied load of 100 g (HVS-1000, Shanghai Jujing Precision Instrument Manufacturing Co., Ltd., Shanghai, China). Ten measurements were made for each specimen to get the average hardness value.

#### 2.2.3. Flexural Strength

Flexural strength of bionic layered composite material was obtained from a three-point bending experiment, which was calculated by Equation (1):

(1) $$\sigma =\frac{3\mathrm{PL}}{2{\mathrm{bh}}^{2}},$$

where *σ* represented the flexure strength. P was the critical load during the bending experiment process. L, b and h were the length of the support span, width and thickness of the samples, respectively.

The homogeneous composites and layered composites with dimensions of 30 mm × 10 mm × 10 mm (Length × Width × Thickness) were tested by a universal testing machine (Model DDL-100, Changchun, China) with the constant loading rate of 0.2 mm/min. The loading span was 24 mm. Average value of flexural strength was calculated from five individual measurements.

#### 2.2.4. Fracture Toughness

Fracture toughness represented the crack arrest property of ceramic reinforced metal matrix composites, which was measured by a single edge notched beam method. Combined with the selected data in Ref. [25], fracture toughness of layered composite was calculated in Equation (2):

(2) $$K_{I}=Y\frac{3\mathrm{PL}}{2{\mathrm{bh}}^{2}}\sqrt{a}.$$

Fracture toughness of samples with dimension of 30 mm × 10 mm × 6 mm (Length × Width × Thickness) was tested by a universal testing machine with a loading rate of 0.2 mm/min (Model DDL-100, Changchun, China). The depth and width of notch was 1.3 mm and 0.2 mm, respectively. The loading span was 24 mm. Under the condition of 0 ≤ a/h ≤ 0.6, the value of Y can be calculated by Equation (3). Values of A_0_, A_1_, A_2_, A_3_ and A_4_ are shown in Table 1. Fracture toughness was obtained from the average value of five parallel tests:

(3) $$Y=A_{0}+A_{1}\frac{a}{h}+A_{2}{\left(\frac{a}{h}\right)}^{2}+A_{3}{\left(\frac{a}{h}\right)}^{3}+A_{4}{\left(\frac{a}{h}\right)}^{4}.$$

#### 2.2.5. Compressive Strength

A universal testing machine (Model DDL-100, Changchun, China) was employed to test compressive strength of the homogeneous and layered composites with dimension of Φ5 mm × 10 mm via Equation (4). The constant loading rate was 0.2 mm/min. The average value of the compression strength was got from five individual tests:

(4) $$\sigma =\frac{P}{A},$$

where *σ*, P and A represented the compression strength, critical load during the compression process and the cross sectional area.

#### 2.2.6. Impact Toughness

A standard Charpy U-notch specimen was used for impact resistance experiment of the sample with dimension of 50 mm × 10 mm × 10 mm (Length × Width × Thickness). The impact testing machine (Model RPK450, Changchun, China) was employed to measured ballistic work in Equation (5). The loading span for impact toughness tests was 40 mm. The average value of the compression strength was obtained from five individual tests:

(5) $$a=\frac{A}{\mathrm{bh}},$$

where a and A represented impact toughness and ballistic work, respectively. b and h were width and thickness of specimen. The corresponding fracture morphologies of mechanical experiments were observed and analyzed via SEM.

## 3. Result and Discussion

### 3.1. Microstructure Characteristics of Bionic Models

**Figure 1:**
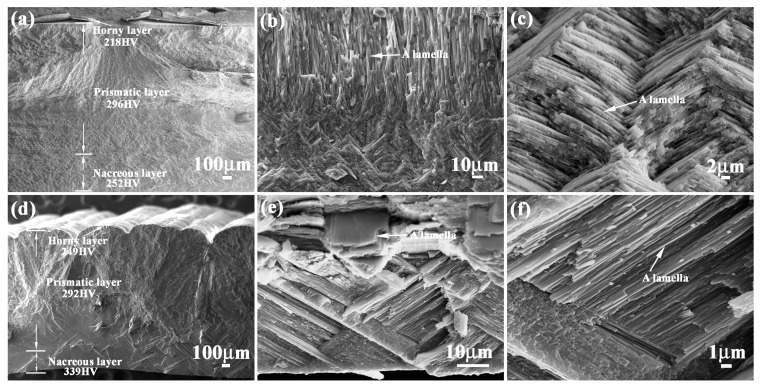
Microstructure of (**a**–**c**) *Meretrix lusoria* shell and (**d**–**f**) *Rapana venosa* shell. (**a**) layered morphology of *Meretrix lusoria* shell; (**b**) microstructure of the conterminous area between prismatic layer and nacreous layer; (**c**) magnified morphology of nacreous layer; (**d**) layered morphology of *Rapana venosa* shell; (**e**) microstructure of the conterminous area between prismatic layer and nacreous layer; (**f**) magnified morphology of nacreous layer.

Figure 1 shows the microstructure characteristics of *Meretrix lusoria* shell and *Rapana venosa* shell. *Meretrix lusoria* shell can be divided into three layers with different microhardness: horny layer (218 HV), prismatic layer (296 HV) and nacreous layer (252 HV), as shown in Figure 1a. The prismatic layer that approached the nacreous layer of *Meretrix lusoria* shell consists of various lamellae, which can be found in Figure 1b. In the nacreous layer of Figure 1c, the extension direction of a lamella crisscrosses the adjacent ones, exhibiting the typical characteristics of a crossed-lamellar structure. Even though the extension direction of lamellae in the prismatic layer is different from that in nacreous layer, the interface exhibits firm connection. The *Rapana venosa* shell in Figure 1d can also be divided into three layers with various microhardness values: horny layer (249 HV), prismatic layer (292 HV) and nacreous layer (339 HV). Compared with Figure 1b, the prismatic layer of *Rapana venosa* shell consists of many lamellae with clintheriform shape, which can be found in the conterminous area between prismatic layer and nacreous layer in Figure 1e. The nacreous layer of Figure 1f also consists of crossed lamellae, conforming to the characteristics of crossed-lamellar structure. Besides different microstructures, *Meretrix lusoria* shell and *Rapana venosa* shell with layered structures in macroscopic and microcosmic views have their own their unique microhardness gradient. In our previous study [23], microhardness played an important role in mechanical properties of shells. Different layers exhibited different hardness values. *Meretrix lusoria* shell and *Rapana venosa* shell present the models of “Relatively hard-Most hard-Relatively soft” and “Relatively soft-Relatively hard-Most hard”, respectively from outer layer to inner layer. The coupling effects of layered microstructure and hard/soft combination model enhance the mechanical properties of two shells. Based on microstructure characteristics, the *Meretrix lusoria* shell and *Rapana venosa* shell provide excellent bionic coupling models for designing composite materials with high mechanical properties.

### 3.2. Design of Bionic Coupling Model

**Figure 2:**
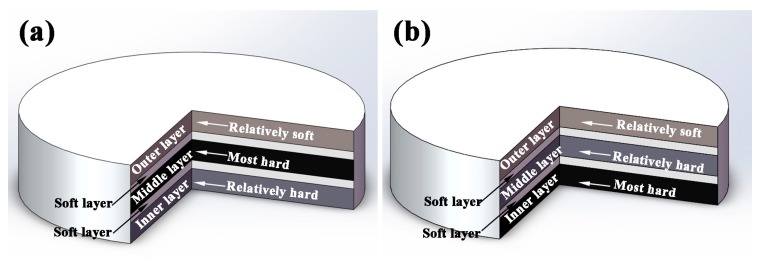
Schematic diagrams of bionic coupling models based on (**a**) *Meretrix lusoria* shell and (**b**) *Rapana venosa* shell.

As two kinds of typical bionic models, the coupling effects between layered structure and the hard/soft combination model in *Meretrix lusoria* shell and *Rapana venosa* shell are simplified, respectively. Figure 2 shows the schematic diagrams of bionic coupling models inspired from *Meretrix lusoria* shell and *Rapana venosa* shell, which are composed of three hard layers including “Relatively soft layer”, “Relatively hard layer” and “Most hard layer” and two soft layers. According to the two microhardness distribution patterns of bionic models in Figure 1, the three hard layers are arranged in sequences of “Relatively soft”, “Most hard” and “Relatively hard” in Figure 2a as well as “Relatively soft”, “Relatively hard” and “Most hard” in Figure 2b from outer layer to inner layer, respectively. Soft layers space and connect contiguous two hard layers, establishing layered structure and the hard/soft combination model of bionic design. Bionic coupling models establish the connection between bionic design and bionic fabrication.

### 3.3. Microstructure and Phase Identification of Bionic Layered Composite Materials

**Figure 3:**
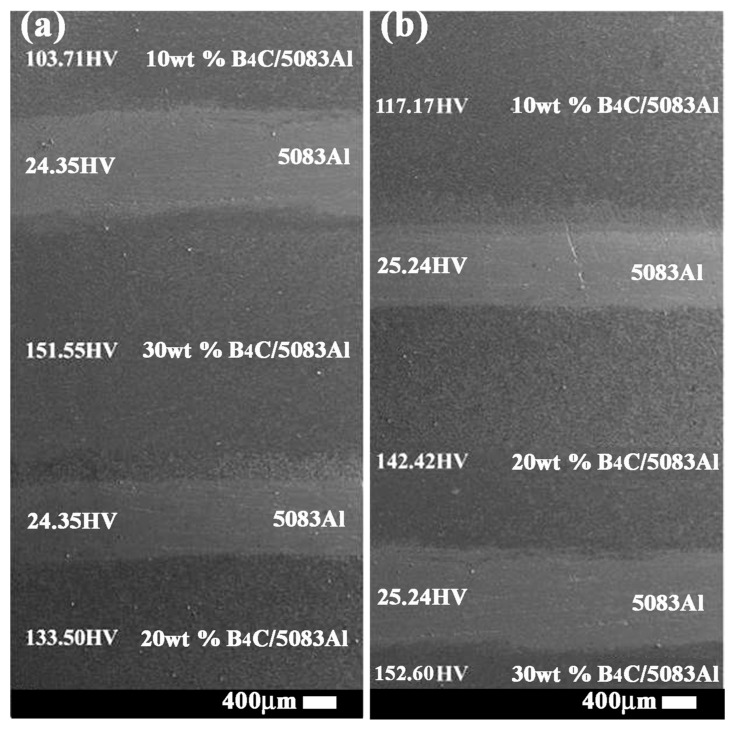
Layered structure and microhardness of (**a**) Bionic *Meretrix lusoria* shell layered composite material and (**b**) Bionic *Rapana venosa* shell layered composite material.

According to the bionic coupling model, the bionic *Meretrix lusoria* shell and *Rapana venosa* shell layered composite materials were fabricated via hot pressed sintering. The layered structure and corresponding hardness are shown in Figure 3a,b. B_4_C reinforced 5083Al matrix composites with different B_4_C contents were treated as hard layers. Pure 5083Al was treated as soft layers. Variation of B_4_C content significantly affected hardness value of hard layers. With the increase of B_4_C content, microhardness of B_4_C/5083Al layers increased gradually. The combination between hard and soft layers realized the characteristics of layered structure and the hard/soft combination model in Figure 2. Hot pressed sintering maintained the high compactness of hard layers, existence of 5083Al layers and firm connection of layered structure. From the point view of morphology, bionic design was accomplished, which provided a material base for investigation of mechanical properties.

**Figure 4:**
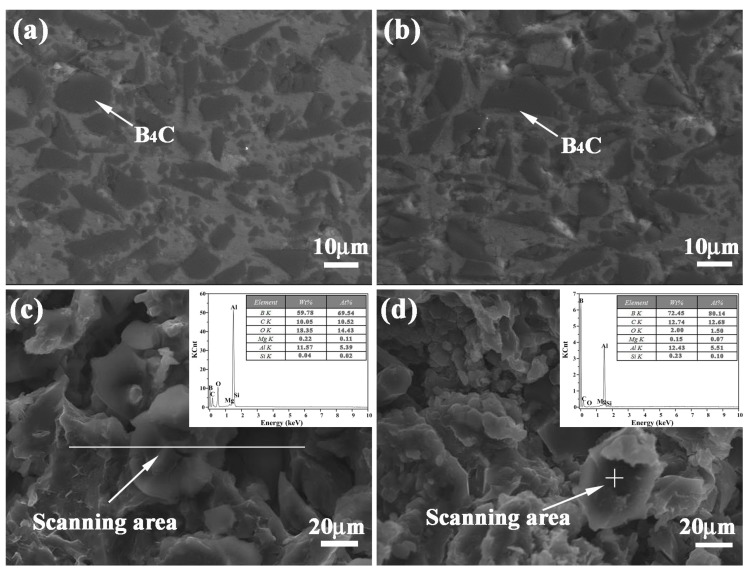
Metallography of inner layers in (**a**) Bionic *Meretrix lusoria* shell and (**b**) *Rapana venosa* shell layered composite materials, fracture morphology and EDS analysis of inner layers in (**c**) Bionic *Meretrix lusoria* shell and (**d**) Bionic *Rapana venosa* shell layered composite materials.

In order to disclose the distribution and bonding patterns of B_4_C particles in a 5083Al matrix, metallography and corresponding fracture morphology were observed. Figure 4a,b exhibit the metallography of inner layers of 20 wt % and 30 wt % B_4_C/5083Al in bionic *Meretrix lusoria* shell composite and bionic *Rapana venosa* shell composite, respectively. B_4_C particles present homogeneous distribution without agglomeration and macrosegregation in the matrix. The interface between B_4_C and 5083Al matrix exhibit firm bonding without any cracks, which proves the practicability of hot pressed sintering.

The fracture morphologies and corresponding EDS analysis of 20 wt % and 30 wt % B_4_C/5083Al layers are shown in Figure 4c,d, respectively. Combined with EDS data, the high weight and atom percentage of B and C confirm the existence and position of B_4_C on the fracture surface. The uniform distribution of ceramic particles and high bonding strength between reinforcement and matrix exhibit the high wettability of B_4_C. Figure 4c indicates that the elements in the line scanning area consists of B, C, O, Mg, Al and Si, which is similar to the point scanning area in Figure 4d. The existence of Mg and Si resulted from the components of Al 5083. Metallography, fracture appearance and EDS results approved the existence of B_4_C ceramic particles from the viewpoint of microstructure. Analysis of fracture morphology confirmed that the B_4_C reinforced 5083Al matrix composite was prepared successfully in every hard layer of bionic layered composites with different bionic models, which built the substantial base of high mechanical property.

**Figure 5:**
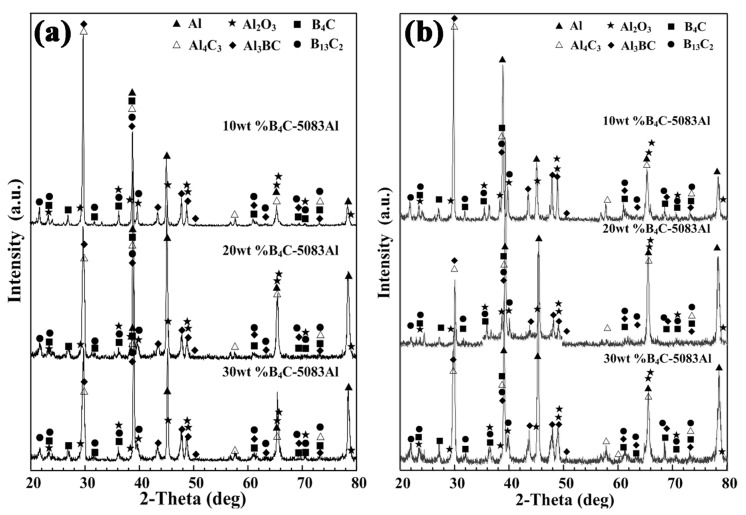
Phase identification of (**a**) bionic *Meretrix lusoria* shell and (**b**) *Rapana venosa* shell layered composite materials.

Figure 5a,b show the phase identification of outer layer, middle layer and inner layer of bionic *Meretrix lusoria* shell and bionic *Rapana venosa* shell layered composite materials, respectively. Besides Al and B_4_C, B_4_C/5083Al layers consist of B_13_C_2_, Al_3_BC, Al_4_C_3_ and Al_2_O_3_, which is similar to the EDS results in Figure 4. A hot pressed sintering method was conducted in air. Part of Al reacted with oxygen, forming the Al_2_O_3_. The existence of B_13_C_2_, Al_3_BC and Al_4_C_3_ enhances bonding strength between ceramic and matrix. The different layered structure maintains the steady phase components. From the viewpoint of phase identification, bionic layered composite materials with different hardness distributions are successfully fabricated in accordance with bionic coupling models of *Meretrix lusoria* shell and *Rapana venosa* shell. Combined with Figure 3, Figure 4 and Figure 5, the fabrication of bionic layered composites proves the feasibility of bionic coupling models. The integrated layered structure, hard/soft combination model and the steady phase components significantly affect mechanical properties of bionic layered composites. Therefore, the corresponding mechanical properties including flexural strength, fracture toughness, compressive strength and impact toughness were investigated.

### 3.4. Mechanical Properties of Bionic Layered Composite Materials

#### 3.4.1. Flexural Strength

**Figure 6:**
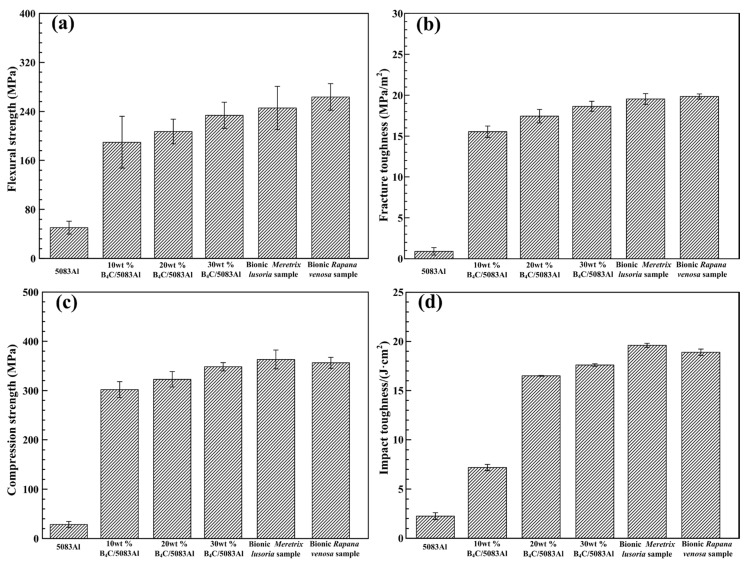
(**a**) Flexural strength; (**b**) Fracture toughness; (**c**) Compression strength and (**d**) Impact toughness values of 5083Al, B_4_C/5083Al composite materials, bionic *Meretrix lusoria* shell and bionic *Rapana venosa* shell layered composite materials.

Flexural strength, fracture toughness, compression strength and impact toughness values of bionic layered composite materials are shown in Figure 6a–d, respectively. Flexure strength values of 5083Al, 10 wt % B_4_C/5083Al, 20 wt % B_4_C/5083Al and 30 wt % B_4_C/5083Al were 51.2, 191.0, 205.3 and 230.5 MPa, respectively. With the increase of B_4_C content, flexure strength of B_4_C/5083Al composites increased. The flexural strength values of bionic *Meretrix lusoria* sample and bionic *Rapana venosa* sample were 249.6 and 169.6 MPa, respectively, which were higher than that of Al matrix and homogeneous composites.

**Figure 7:**
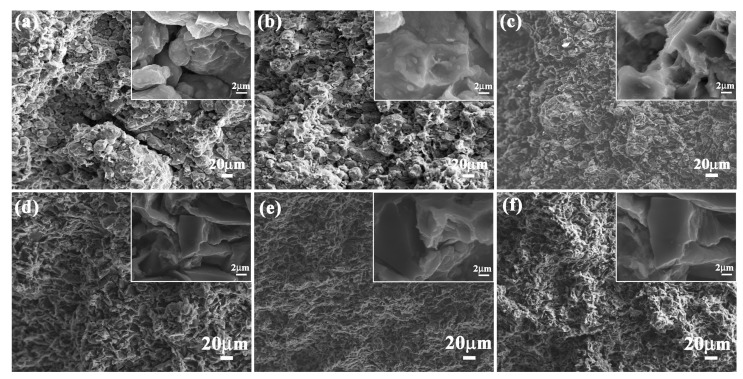
Flexural appearance of (**a**) Outer layer; (**b**) Middle layer and (**c**) Inner layer of bionic *Meretrix lusoria* shell composite material and (**d**) Outer layer; (**e**) Middle layer and (**f**) Inner layer of bionic *Rapana venosa* shell composite material.

To investigate the effect of layered structure and hard/soft combination model on flexural strength of bionic composites, the flexural appearance was observed. The corresponding results are shown in Figure 7. The three hard layers of bionic *Meretrix lusoria* composite material exhibited different morphology characteristics. Some long and wide cracks appeared on the outer layer (Figure 7a). Some small cracks existed on the middle layer (Figure 7b). There was no existence of continuous and destructive cracks on the inner layer (Figure 7c). The three hard layers of a bionic *Rapana venosa* sample presented relative smooth flexural surfaces. Some small cracks with different extension directions can also be found in the outer layer, middle layer and inner layer, as shown in Figure 7d–f. In the magnified microstructure of the two bionic composites, a number of pits appeared on flexural appearance. Under the applied bend force, the outer layer endured the highest applied load, resulting in the existence of cracks. The existence of soft 5083Al layers absorbed load and reduced damage of middle and inner layers. Therefore, no destructive cracks can be found in inner layers. The intergranular fracture mechanism led to the existence of the pit in the flexural appearances. Combined with Figure 6, layered structure and hard/soft combination model significantly enhance flexural strength of bionic layered composites.

#### 3.4.2. Fracture Toughness

**Figure 8:**
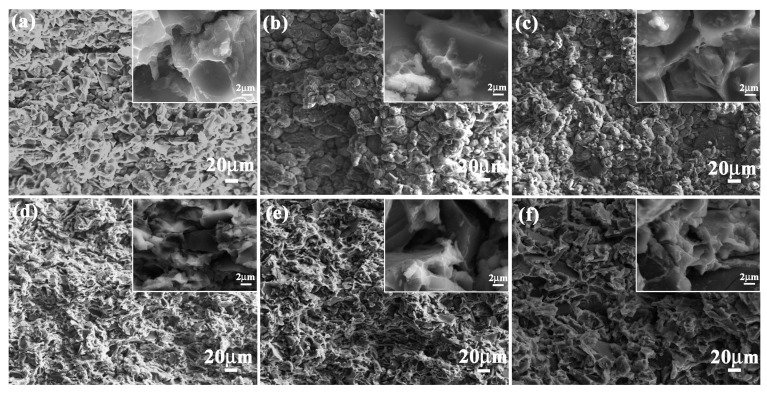
Fracture appearance of (**a**) Outer layer; (**b**) Middle layer and (**c**) Inner layer of bionic *Meretrix lusoria* shell composite material and (**d**) Outer layer; (**e**) Middle layer and (**f**) Inner layer of bionic *Rapana venosa* shell composite material.

As an important performance parameter, fracture toughness evaluated the crack arrest property of ceramic composite materials. Fracture toughness values of 5083Al, 10 wt % B_4_C/5083Al, 20 wt % B_4_C/5083Al and 30 wt % B_4_C/5083Al were 2.3, 14.1, 16.5 and 17.7 MPa/m^2^, respectively, as shown in Figure 6b. The bionic *Meretrix lusoria* shell and bionic *Rapana venosa* shell layered composite materials owned the higher fracture toughness of 19.6 and 20.5 MPa/m^2^, respectively. In order to understand the crack arrest mechanism of the two bionic layered composite materials, the corresponding fracture appearances were investigated, as shown in Figure 8a–f. From the morphologies and megascopic images, it can be found that fracture appearance of the three layers of the two bionic layered composite materials were characterized by a number of small pits. Under the influence of applied load, new cracks appeared in the crack predefined position, and extended into the inner parts of bionic layered composites. During the process of crack extension, most B_4_C particles in hard layers were extracted, causing intergranular fracture phenomenon and irregular morphology. As the connection layers, soft layers of 5083Al in two kinds of bionic layered composites restricted crack extension and enhanced fracture toughness values, resulting from the intercoupling effect of layered structure and hard/soft combination model.

#### 3.4.3. Compressive Strength

**Figure 9:**
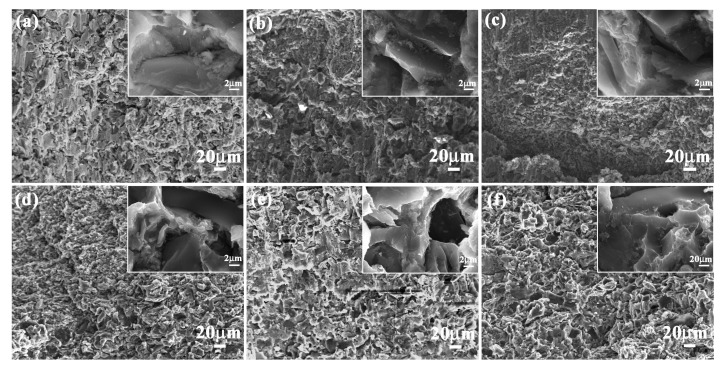
Compression appearance of (**a**) Outer layer; (**b**) Middle layer and (**c**) Inner layer of bionic *Meretrix lusoria* shell composite material and (**d**) Outer layer; (**e**) Middle layer and (**f**) Inner layer of bionic *Rapana venosa* shell composite material.

Compression strength values of bionic *Meretrix lusoria* shell (366.2 MPa) and bionic *Rapana venosa* shell (355.4 MPa) layered composite materials were higher than those of 5083Al (30.5 MPa), 10 wt % B_4_C/5083Al (300.7 MPa), 20 wt % B_4_C/5083Al (325.6 MPa) and 30 wt % B_4_C/5083Al (340.4 MPa), respectively, which can be found in Figure 6c. Bionic layered composites possessed the highest compressive strength, exhibiting perfect compression resistance. Compression appearances and corresponding magnified microstructures of the three hard layers in the two kinds of bionic layered composite are shown in Figure 9a–f. A large number of cracks and pits resulted from intergranular fracture phenomenon of B_4_C particles were presented on irregular compression appearance. Moreover, some cracks extended across the outer layer and middle layer in a bionic *Meretrix lusoria* shell composite. The broken ceramics, spalling fragments and pits on magnified compression appearances presented the anti-compression ability. The broken B_4_C particles resulted in pits and cracks to defuse load, which indicated the dominate fracture behavior of intergranular fracture. Soft layers of 5083Al with high toughness reset extension direction of cracks in the two kinds of layered structures. The combination of hard layers and soft layers led to the higher compression strength than that of homogenous composites.

#### 3.4.4. Impact Toughness

**Figure 10:**
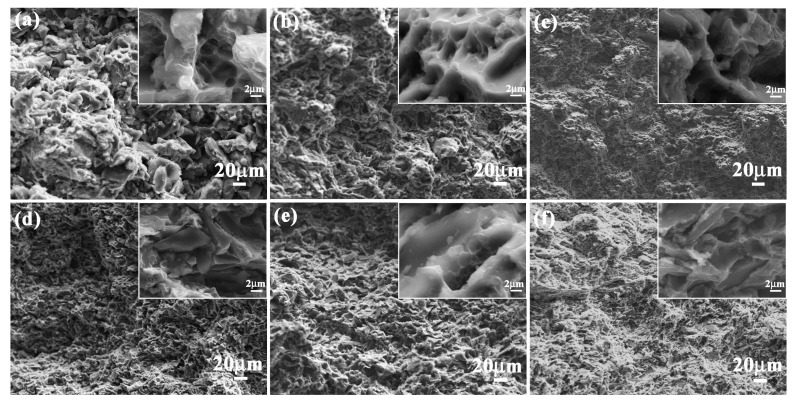
Impact appearance of (**a**) Outer layer; (**b**) Middle layer; and (**c**) Inner layer of bionic *Meretrix lusoria* shell composite material and (**d**) Outer layer; (**e**) Middle layer; and (**f**) Inner layer of bionic *Rapana venosa* shell composite material.

Impact toughness values of bionic *Meretrix lusoria* shell and bionic *Rapana venosa* shell layered composite materials in Figure 6d were 20.5 J/cm^2^ and 19.6 J/cm^2^, respectively, which were higher than those of B_4_C/5083Al composites. Variation of B_4_C content influenced the impact toughness of B_4_C/5083Al composites. Impact toughness of 5083Al, 10 wt % B_4_C/5083Al, 20 wt % B_4_C/5083Al and 30 wt % B_4_C/5083Al were 3.2, 7.0, 15.3 and 16.9 J/cm^2^, respectively. Figure 10a–f were the impact appearance of three hard layers in bionic layered composites with two coupling models. The impact appearances were characteristic by a large number of pits. Several dimples resulted from a certain degree of plastic deformation appear on the impact fracture surfaces. Moreover, bionic layered composites exhibited high interlayer bonding strength between hard layers and soft layers.

Combined with Figure 6, Figure 7, Figure 8, Figure 9 and Figure 10, bionic layered composite materials with *Meretrix lusoria* shell and *Rapana venosa* shell intercoupling models owned relative high values of flexural strength, fracture toughness, compression strength and impact toughness, which built the material base for practical applications. The hard layers in bionic layered composites absorbed the applied load in the form of intergranular fracture. Besides connection role, the soft layers with high toughness in bionic layered composites restrained the slabbing phenomenon in the layered structure. Moreover, soft layers owned the property of resetting extension direction of cracks among layers. The intercoupling effect of layered structure and hard/soft combination model played the key role in the high mechanical properties of bionic composites, which proved the feasibility and practicability of bionic design.

## 4. Conclusions

Based on microstructure characteristics of *Meretrix lusoria* shell and *Rapana venosa* shell, two kinds of bionic coupling models were established to guide the design and fabrication of bionic coupling layered B_4_C/5083Al composite materials, respectively. Attributed to the coupling between layered structure and hard/soft combination model, the bionic composite materials owned high mechanical properties including flexural strength, fracture toughness, compressive strength and impact toughness:

(1) *Meretrix lusoria* shell and *Rapana venosa* shell can be divided into three layers with different hardness. *Meretrix lusoria* shell and *Rapana venosa* shell presented the models of “Relatively hard-Most hard-Relatively soft” and “Relatively soft-Relatively hard-Most hard”, respectively, from outer layer to inner layer. The coupling between layered microstructure and hard/soft combination model enhanced the mechanical properties of two shells.
(2) According to characteristics of two shells, two kinds of bionic coupling models were built to guide the design of the bionic layered composites. B_4_C/5083Al composites with different B_4_C contents were treated as hard layers. Pure 5083Al was treated as soft layers. Hot pressed sintering maintained advantages of bionic models and realized the transformation from bionic design to bionic fabrication.
(3) The layered structure and different hardness distribution patterns of bionic composites maintained the steady microstructure and phase components of B_4_C/5083Al composites, proving the validity of bionic coupling models and building material base for realization of high mechanical properties.
(4) Compared with the homogenous B_4_C/5083Al composites, bionic layered composites exhibited higher flexural strength, fracture toughness, compressive strength and impact resistance. The intercoupling effect of layered structure and hard/soft combination model was the key point for the property of bionic composites. The fabrication of bionic coupling layered composites proved the feasibility and practicability of bionic design and provided a new idea and method for the fabrication of a ceramic/Al composite being lightweight and having a high mechanical strength.

## Figures and Tables

**Table 1:** Value of A_0_, A_1_, A_2_, A_3_ and A_4_ [25].

Loading Mode	A_0_	A_1_	A_2_	A_3_	A_4_
Three-point bending (L/h = 4)	1.93	−3.07	14.53	−25.11	25.80
